# BIIB021, an Hsp90 inhibitor, effectively kills a myelodysplastic syndrome cell line via the activation of caspases and inhibition of PI3K/Akt and NF-κB pathway proteins

**DOI:** 10.3892/etm.2014.1651

**Published:** 2014-03-28

**Authors:** SHENGYUN LIN, JING LI, WENJING ZHOU, WENBIN QIAN, BO WANG, ZHI CHEN

**Affiliations:** 1 Department of Hematology, The First Affiliated Hospital, Zhejiang Chinese Medical University, Hangzhou, Zhejiang 310006, P.R. China; 2 Institute of Hematology, The First Affiliated Hospital, School of Medicine, Zhejiang University, Hangzhou, Zhejiang 310003, P.R. China

**Keywords:** BIIB021, myelodysplastic syndrome cell line, caspases, PI3K/Akt, NF-κB pathway proteins

## Abstract

The novel orally available inhibitor of the molecular chaperone heat shock protein 90 (Hsp90), BIIB021, induces the apoptosis of various types of tumor cell *in vitro* and *in vivo*. However, the effects and mechanisms of this agent on myelodysplastic syndrome (MDS) cell lines remain unknown. The aim of this study was to investigate the effects of BIIB021 on SKM-1 cells (a MDS cell line) and examine its mechanisms of action. The results showed that BIIB021 inhibited the growth of SKM-1 cells effectively *in vitro*. The treatment of SKM-1 cells with BIIB021 resulted in the inhibition of cell growth through G0/G1-phase cell cycle arrest and induced apoptosis by activating caspase-3, -8 and -9. Furthermore, this study also demonstrated that the mechanisms of apoptosis in SKM-1 cells were associated with the suppression of the phosphatidylinositide 3-kinase/Akt and nuclear factor-κB signaling pathways. Therefore, the findings indicate a novel approach for the treatment of high-risk MDS.

## Introduction

Myelodysplastic syndrome (MDS) is a clinically and cytogenetically heterogeneous group of clonal diseases characterized by ineffective hematopoiesis-associated cytopenias, consequent bleeding, infections and a high risk of acute myeloid leukemia (AML) transformation (1,2). The International Prognostic Scoring System (IPSS) for MDS is based upon weighted data on the bone marrow blast percentage, cytopenia and cytogenetics and it separates patients into four prognostic groups: Low, intermediate-1, intermediate-2 and high. The median overall survival time of patients with high-risk MDS (commonly defined as the patients with an IPSS risk score >1.0) is approximately 14 months (3). These patients have low remission rates and short periods of disease-free survival despite chemotherapy (4–6). MDS occurs more frequently in older individuals. Currently, there is no curative treatment for MDS, with the exception of allogenic stem cell transplantation which is unsuitable for the majority of elderly patients due to comorbid illness or poor performance status. Therefore, elderly patients generally receive low-dose chemotherapy, supportive care or investigational treatment (2,7). The general view is that complete or partial remission is a prerequisite for the prolonged survival of patients with high-risk MDS and it is achieved using chemotherapy regimens similar to those used for AML (8). However, traditional chemotherapies for MDS have limited success rates, so it is necessary to explore novel therapeutic targets and agents that have higher selectivity for tumor cells and less toxicity toward normal tissues.

Heat shock protein 90 (Hsp90) is attractive molecular target as it acts as a chaperone that prevents the degradation of a number of important cellular oncoproteins, including receptor and nonreceptor kinases (9). The overexpression of Hsp90 in acute leukemia cells has been confirmed by several studies (10,11). Furthermore, the expression levels of Hsp90 are higher in blastic MDS, which is associated with poor prognosis (12,13). Hsp90 inhibitory agents, including the ansamycin antibiotic geldanamycin, bind to the ATP-binding pocket of Hsp90, thereby disrupting Hsp90 function, and thus present as promising drugs for the treatment of cancer (14). Phase I/II clinical trials of Hsp90 inhibitors have been conducted, including a Phase I trial of the Hsp90 inhibitor tanespimycin (17-AAG) in relapsed and refractory acute leukemia (15,16).

BIIB021 was the first *‘*fully synthetic’ Hsp90 inhibitor to be used for the clinical treatment of solid tumors and hematological malignancies (17,18). BIIB021 induces the degradation of Hsp90 client proteins, including human epidermal growth factor receptor-2 (HER-2), Akt and RAF proto-oncogene serine/threonine-protein kinase (Raf-1), and results in tumor growth inhibition (18). A phase II clinical trial (19) showed that BIIB021 improves the outcome of patients with gastrointestinal stromal tumors refractory to imatinib and sunitinib. Despite a broad prospect for further clinical application of this agent, no studies have been conducted using MDS cells. The present study focused on the therapeutic effects and mechanisms of one molecularly targeted agent, the Hsp90 inhibitor BIIB021, on high-risk MDS *in vitro*.

## Methods and materials

### Cell culture and reagents

SKM-1 cells (JCRB0118; Japanese Collection of Research Bioresources Cell Bank, Osaka, Japan) were cultured in RPMI-1640 medium (Gibco, Grand Island, NY, USA) with 10% fetal bovine serum (Gibco) at 37°C in a humidified atmosphere of 5% CO_2_. BIIB021 was purchased from Selleck Chemicals (Houston, TX, USA). Methylcellulose and 3-(4,5-dimethylthiazol-2-yl)-2,5-diphenyltetrazolium bromide (MTT) were purchased from Sigma-Aldrich (St. Louis, MO, USA). All antibodies used in the western blot analysis were purchased from Cell Signaling Technology, Inc. (Danvers, MA, USA), with the exception of human anti-β-actin. Insulin-like growth factor-1 (IGF-1) was purchased from Peprotech (Rocky Hill, NJ, USA). The caspase inhibitors z-IETD-fmk and z-LEHD-fmk were purchased from Biovision (Palo Alto, CA, USA).

### Cell viability assay

To evaluate the effects of BIIB021 on the MDS cells, MTT assays were performed. The SKM-1 cells were cultured at a density of 5×10^4^ cells/well in a 96-well plate and treated with BIIB021 at concentrations of 50, 100, 200 and 400 nM, respectively. Following 24 or 48 h incubation, MTT was added to each well and the plates were incubated for an additional 4 h at 37°C. The supernatant was removed, followed by the addition of 200 μl dimethyl sulfoxide (Amresco, Solon, OH, USA). The absorbance at a wavelength of 570 nm was detected with an enzyme-linked immunosorbent assay plate reader (Bio-Rad, Hercules, CA, USA). Each assay was performed three times in triplicate.

### Annexin V binding assay

The cells were cultured at a density of 5×10^4^ cells/well in a six-well plate and treated for 24 h with BIIB021 at concentrations of 0, 100, 200 and 400 nM. After 24 h of treatment at 37°C, the cells were collected and washed. Aliquots of the cells were resuspended in 500 μl binding buffer and stained with 5 μl Annexin V-fluorescein isothiocyanate (FITC) and 5 μl propidium iodide (PI; Biouniquer Technology Co., Ltd., Suzhou, China) for 15 min in the dark, and examined by flow cytometry. Data acquisition and analysis were performed on a FACSCalibur flow cytometer (Becton-Dickinson, Franklin Lakes, USA) using CellQuest software (Becton-Dickinson).

### Cell cycle analysis

The cells were treated with BIIB021 at concentrations of 0, 100 and 200 nM and incubated for 24 h at 37°C. The cells were harvested, washed twice with cold phosphate-buffered saline (PBS), and suspended and fixed in 75% ice-cold ethanol overnight at 4°C. Subsequently, the sample was washed with PBS and incubated with 250 μg/ml RNase A and 10 μg/ml PI for 30 min. The cells were analyzed using a FACSCalibur flow cytometer.

### Hoechst 33258 DNA staining

To detect the morphological changes following treatment with BIIB021 (0, 100, 200 and 400 nM), SKM-1 cells were plated at an initial density of 1×10^5^ cells/well in a 24-well plate and treated with BIIB021 for 24 h. The treated cells were fixed with 3.7% paraformaldehyde for 30 min and then stained with Hoechst 33258 (0.5 μg/ml) for 20 min at room temperature. The cells were counted under an Axiovert fluorescence microscope (Carl Zeiss, Göttingen, Germany) with an excitation wavelength of 350 nm and an emission wavelength of 460 nm.

### Western blot analysis

The cells were harvested 24 h after treatment at the indicated doses and times, and the cell lysates were subjected to western blotting, performed as described previously (20). Briefly, the cells were collected and lysed using 10 mM Tris, 1 mM ethylenediaminetetraacetic acid, 10 mM KCl and 0.3% Triton X-100 (pH 7.9). The concentration of the protein samples was measured by the Bradford method. The protein samples were separated by sodium dodecyl sulfate-polyacrylamide gel electrophoresis (SDS-PAGE) and then electroblotted onto Hybond-P polyvinylidene fluoride membranes (Amersham, Piscataway, NJ, USA). The membranes were subjected to western blot analysis with primary antibodies to caspase-8, -9 and -3, poly(ADP-ribose) polymerase (PARP), p110δ, Akt, phospho-Akt, p65, phospho-p65, cyclin-dependent kinase (CDK)4, CDK6, cyclin D1 and β-actin (Sigma-Aldrich). The secondary antibodies used in this study were provided by Multisciences Co., Ltd. (Hangzhou, China).

### Statistical analysis

Experimental results are statistically presented as the mean ± standard deviation. Data were analyzed by one-way analysis of variance. P<0.05 was considered to indicate a statistically significant difference.

## Results

### Effects of BIIB021 on the viability of the human MDS cell line

**Figure 1:**
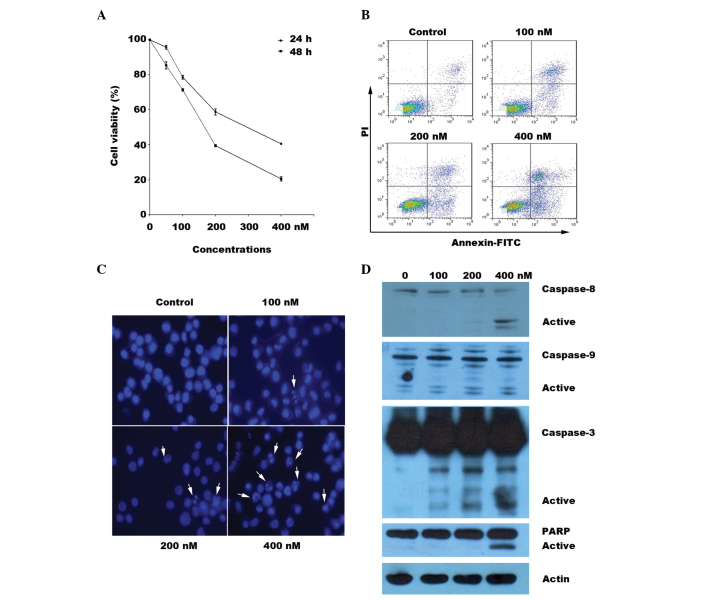
BIIB021 inhibits cellular viability and induces apoptosis in SKM-1 cells. (A) After 24 and 48 h treatment with BIIB021 (50, 100, 200 and 400 nM), the cells were incubated with MTT to determine the levels of cell proliferation. The data shown are the mean ± SD of three independent experiments. (B) The SKM-1 cells were treated with BIIB021 at the indicated concentrations for 24 h and processed for Annexin V-FITC and PI double staining. The apoptotic cells were then quantitatively monitored. (C) Followng a treatment similar to that in (B), the nuclear morphology of the SKM-1 cells as analyzed by Hoechst 33258 staining. The arrows indicate apoptotic nuclei. (D) Cell lysates were prepared from SKM-1 cells following incubation with or without BIIB021 at the indicated concentrations for 24 h. Equal amounts of proteins per sample were resolved by SDS-PAGE and then transferred to a PVDF membrane, which was probed for the expression levels of caspase-3, -8 and -9, PARP and β-actin. PI, propidium iodide; FITC, fluorescein isothiocyanate; PARP, poly(ADP-ribose) polymerase; MTT, 3-(4,5-dimethylthiazol-2-yl)-2,5-diphenyltetrazolium bromide; SDS-PAGE, sodium dodecyl sulfate-polyacrylamide gel electrophoresis; PVDF, polyvinylidene fluoride.

The SKM-1 cells were tested for sensitivity to BIIB021 in a cell proliferation assay. The 50% inhibitory concentration (IC_50_) values were 275.2 nM for 24 h and 163.9 nM for 48 h. As shown in Fig. 1A, BIIB021 effectively inhibited SKM-1 cell growth in a concentration- and time-dependent manner. The Annexin V binding assay confirmed that BIIB021 induced SKM-1 cell apoptosis in a concentration-dependent manner (Fig. 1B). Morphological observation by Hoechst 33258 DNA staining showed an increased number of cells with nuclear condensation and fragmentation following treatment with BIIB021 for 24 h (Fig. 1C).

### BIIB021 induces SKM-1 cell apoptosis via activation of the caspase family

**Figure 2:**
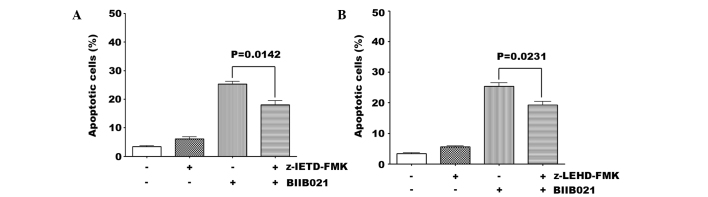
Caspase-8 and -9 inhibitors partially inhibit BIIB021-mediated apoptosis. The SKM-1 cells were preincubated with 20 μM (A) z-IETD-FMK or (B) z-LEHD-FMK for 1 h prior to the treatment with 400 nM BIIB021 for 24 h. Subsequently, the effects of the specific caspase inhibitors on BIIB021-induced apoptosis were evaluated by Annexin V/PI double staining. PI, propidium iodide.

To detect the mechanisms of BIIB021-induced cell apoptosis in the MDS cells, western blotting was used to measure the levels of activation of the caspase family. BIIB021 triggered concentration-dependent cleavage of caspase-3, -8 and -9 and PARP (Fig. 1D). Furthermore, the effect of caspase inhibitors on BIIB021-induced apoptosis was observed. The caspase-8 (20 μM) and caspase-9 (20 μM) inhibitors partially inhibited BIIB021-induced apoptosis (Fig. 2). These results suggest that caspase-8 and -9 inhibitors are able to attenuate BIIB021-induced apoptosis, which indicates that BIIB021 caused apoptosis through activating the cascade to the caspase-8 and -9 pathways in SKM-1 cells.

**Figure 3:**
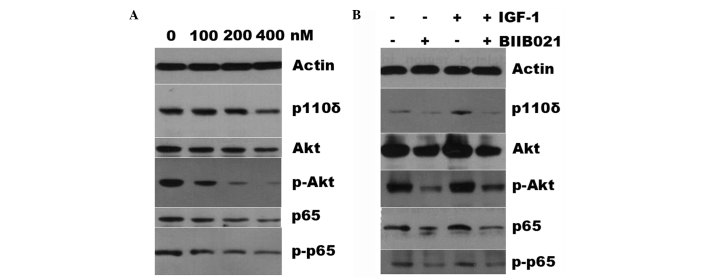
BIIB021 reduces the expression levels of the PI3K/Akt and NF-κB pathway proteins, and IGF-1 does not attenuate BIIB021-mediated inhibition of these proteins. (A) The SKM-1 cells were treated with different concentrations (100, 200 and 400 nM) of BIIB021 for 24 h, and the total proteins were extracted and subjected to western blot analysis using primary antibodies for p110δ, Akt, phospho-Akt, p65 and phospho-p65. Actin was used as a protein loading control. (B) The SKM-1 cells were treated with or without BIIB02 (400 nM), IGF-1 (100 ng/ml), or BIIB021 combined with IGF-1. Whole cell lysates were then subjected to SDS-PAGE followed by immunoblotting with the antibody that recognizes the corresponding antigens. PI3K, phosphatidylinositide 3-kinase; NF-κB, nuclear factor-κB; IGF-1, insulin-like growth factor-1; SDS-PAGE, sodium dodecyl sulfate-polyacrylamide gel electrophoresis.

BIIB021 inhibits the phosphatidylinositide 3-kinase (PI3K)/Akt and nuclear factor (NF)-κB signaling pathways. It has been demonstrated that the PI3K/Akt and NF-κB signaling pathways are activated in high-risk MDS patients and activation of the signaling pathways is responsible for the suppression of apoptosis of MDS cells, contributing to AML transformation (21–24). Furthermore, lasting activation of NF-κB results in drug resistance (25). Therefore, the present study tested whether the mechanisms of BIIB021-induced cell apoptosis are involved in the PI3K/AKT and NF-κB signaling pathways. Fig. 3A shows that the protein expression levels of the PI3K isoforms p110δ, p65 and Akt were only slightly reduced in the SKM-1 cells treated with BIIB021, whereas BIIB021 markedly reduced the levels of phospho-Akt and phospho-p65 in a concentration-dependent manner. Subsequently, whether the downregulation of the levels of these proteins was reversed by IGF-1 was examined. As shown in Fig. 3B, pretreatment of the SKM-1 cells with 100 ng/ml IGF-1 did not attenuate the BIIB021-mediated suppression of the protein expression, despite the fact that IGF-1 alone upregulated the expression levels of p110, p65 and Akt and of phospho-Akt and phospho-p65.

### Inhibition of SKM-1 cell proliferation is due to G0/G1 arrest via the regulation of cell cycle-related proteins

**Figure 4:**
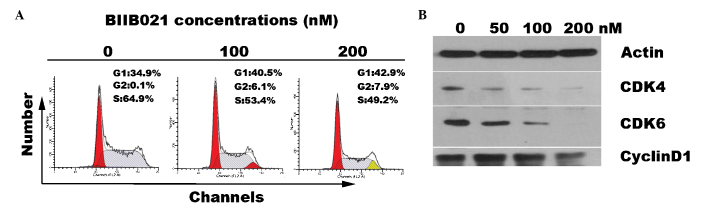
Effects of BIIB021 on the cell cycle in SKM-1 cells are associated with the inhibition of cellular proteins, including CDK4/6 and cyclin D1. (A) Following the BIIB021 treatment, the SKM-1 cells were stained with PI and fixed with ethanol. The cell cycle phase was determined by flow cytometric analysis. The data represent one of three independent experiments with the same results. (B) The cells were treated with BIIB021 at the indicated concentrations for 24 h, whole cell extracts were prepared and western blot analysis was performed using specific antibodies. CDK, cyclin-dependent kinase; PI, propidium iodide.

A previous study showed that a Hsp90 inhibitor induced cell cycle arrest at G2/M (26). On the basis of this, the effects of BIIB021 treatment on the cell cycle were examined by DNA flow cytometry in the present study. The results showed that, compared with that of the untreated cells, the number of cells in the G1 phase was markedly increased in the BIIB021-treated group (34.9 versus 40.5 and 42.9% at 100 and 200 nM, respectively). In addition, treatment with BIIB021 resulted in a reduction in the percentage of cells in the S phase (Fig. 4A). These data suggest that BIIB021 inhibits S phase entry. It has been reported that the active complex of CDK4/cyclin D1 allows the release of E2F transcription factors that activate G1/S phase gene expression. In the present study, the results indicated that BIIB021 markedly reduced the levels of CDK4 and CDK6 in the MDS cells, but the inhibitory effect on cyclin D1 was modest (Fig. 4B).

## Discussion

Overexpression of Hsp90 has been observed in a variety of types of cancer (27). Previous studies have indicated the possible role of Hsp90 in MDS pathogenesis and evolution (12,28). For example, it has been reported that Hsp90 is overexpressed in high-risk MDS, as compared with the levels in low-risk MDS and normal bone marrow, and is associated with a poor prognosis for patients (12). Certain Hsp90 inhibitors, including 17-AAG, are used as targeted therapies for cancer as they showed anticancer activity in early clinical trials (17,29,30). BIIB021 has an improved pharmacological profile compared with that of 17-AAG and other Hsp90 inhibitors, particularly with regard to availability through chemical synthesis, metabolic stability, water solubility and ease of administration via oral and intravenous routes (18,31). The present study of BIIB021 shows that the MDS cell line SKM-1 was blocked in the G1 phase of the cell cycle and underwent apoptosis when treated with BIIB021. The IC_50_ values of BIIB021 required to inhibit the growth of the SKM-1 cells were 355.69 and 168.6 nM at 24 h and 48 h, respectively, which is consistent with a previous study indicating that the IC_50_ values of BIIB021 in various types of solid tumor are in the range of 60 to 310 nM (32). The activation of caspase-8 and -9, followed by the downstream activation of caspase-3 and PARP was observed in the BIIB021-treated MDS cells in the present study. Furthermore, caspase-8 and -9 inhibitors partially attenuated the BIIB021-induced apoptosis. These results suggest that two main pathways of procaspase activation (extrinsic death receptor pathway and intrinsic mitochondrial pathway) are involved in the BIIB021-induced apoptosis of MDS cells.

The PI3K/Akt signaling cascade represents one of the major survival pathways that is deregulated in numerous types of cancer and contributes to tumor pathogenesis and therapy resistance. Constitutive activation of the PI3K/Akt signaling pathway and NF-κB is a feature of patients with high-risk MDS (21,33,34). In the present study, it was identified that BIIB021 slightly downregulated the expression levels of p110δ and Akt. Notably, the drug markedly inhibited the phosphorylation of Akt. Marked downregulation of the levels of p65 and phospho-p65 expression were also observed, suggesting that BIIB021 inhibited the activation of NF-κB, which is a downstream regulator of the PI3K/Akt signaling pathway. As constitutive IGF-1/IGF-1 receptor (IGF-1R) signaling contributes to deregulated PI3K activity and overexpression of IGF-1R was observed in malignant clonal cells in bone marrow of MDS in previous studies (35,36), the effects of exogenous IGF-1 stimulation on BIIB021-mediated inhibition of the PI3K/Akt signaling pathways were tested in the present study. It was observed that IGF-1 at 100 ng/ml increased the expression levels of p110δ, phospho-Akt and phospho-p65, suggesting that IGF-1 activates the PI3K/Akt signaling pathways in MDS cells. This result is consistent with the evidence that IGF-1 is a strong PI3K activator (37). However, exogenous IGF-1 stimulation did not abrogate the capacity of BIIB021 to inhibit Akt and NF-κB. Collectively, inhibition of PI3K/Akt and NF-κB contributes to BIIB021-induced apoptosis, implying that BIIB021 may be useful for overcoming drug resistance.

Deregulation of the cell cycle pathway is a contributor to the pathogenesis of MDS (38,39). For example, cyclin D1 levels are increased in high-risk MDS, thereby increasing the proliferation of leukemia (40,41). BIIB021 has a high binding affinity for Hsp90 and consequently inhibits the chaperone activity of Hsp90 and results in degradation of the client proteins (18,41). In the present study, in addition to the effect of G1 cell cycle arrest, BIIB021 was demonstrated to inhibit the expression of CDK4, CDK6 and cyclin D1, which are also Hsp90 client proteins, in MDS cells. This effect of BIIB021 on the cell cycle pathway is similar to that observed in other types of cancer (18).

In summary, the present study demonstrates for the first time, to the best of our knowledge, that BIIB021 has marked activity against MDS cells. Furthermore, BIIB021 causes the degradation of several client proteins, including Akt, CDK4 and CDK6 in MDS cells. Also, NF-κB activity was inhibited in the SKM-1 cells upon treatment with BIIB021 at low nanomolar concentrations. Therefore, BIIB021 is potentially useful for clinical therapy in the treatment of high-risk MDS.

## References

[1] Cogle CR, Craig BM, Rollison DE, List AF (2011). Incidence of the myelodysplastic syndromes using a novel claims-based algorithm: high number of uncaptured cases by cancer registries. Blood.

[2] Tefferi A, Vardiman JW (2009). Myelodysplastic syndromes. N Engl J Med.

[3] Komrokji RS, Corrales-Yepez M, Al Ali N (2012). Validation of the MD Anderson Prognostic Risk Model for patients with myelodysplastic syndrome. Cancer.

[4] Sekeres MA, Schoonen WM, Kantarjian H, List A, Fryzek J, Paquette R, Maciejewski JP (2008). Characteristics of US patients with myelodysplastic syndromes: results of six cross-sectional physician surveys. J Natl Cancer Inst.

[5] Fukumoto JS, Greenberg PL (2005). Management of patients with higher risk myelodysplastic syndromes. Crit Rev Oncol Hematol.

[6] Gore SD, Hermes-DeSantis ER (2009). Enhancing survival outcomes in the management of patients with higher-risk myelodysplastic syndromes. Cancer Control.

[7] Szmigielska-Kapłon A, Robak T (2011). Hypomethylating agents in the treatment of myelodysplastic syndromes and myeloid leukemia. Curr Cancer Drug Targets.

[8] Garcia-Manero G (2011). Treatment of higher-risk myelodysplastic syndrome. Semin Oncol.

[9] Kamal A, Thao L, Sensintaffar J, Zhang L, Boehm MF, Fritz LC, Burrows FJ (2003). A high-affinity conformation of Hsp90 confers tumour selectivity on Hsp90 inhibitors. Nature.

[10] Yufu Y, Nishimura J, Nawata H (1992). High constitutive expression of heat shock protein 90 alpha in human acute leukemia cells. Leuk Res.

[11] Flandrin P, Guyotat D, Duval A, Cornillon J, Tavernier E, Nadal N, Campos L (2008). Significance of heat-shock protein (HSP) 90 expression in acute myeloid leukemia cells. Cell Stress Chaperones.

[12] Duval A, Olaru D, Campos L, Flandrin P, Nadal N, Guyotat D (2006). Expression and prognostic significance of heat-shock proteins in myelodysplastic syndromes. Haematologica.

[13] Flandrin-Gresta P, Solly F, Aanei CM (2012). Heat Shock Protein 90 is overexpressed in high-risk myelodysplastic syndromes and associated with higher expression and activation of Focal Adhesion Kinase. Oncotarget.

[14] Didelot C, Lanneau D, Brunet M, Joly AL, De Thonel A, Chiosis G, Garrido C (2007). Anti-cancer therapeutic approaches based on intracellular and extracellular heat shock proteins. Curr Med Chem.

[15] Kaufmann SH, Karp JE, Litzow MR (2011). Phase I and pharmacological study of cytarabine and tanespimycin in relapsed and refractory acute leukemia. Haematologica.

[16] Jhaveri K, Taldone T, Modi S, Chiosis G (2012). Advances in the clinical development of heat shock protein 90 (Hsp90) inhibitors in cancers. Biochim Biophys Acta.

[17] Taldone T, Gozman A, Maharaj R, Chiosis G (2008). Targeting Hsp90: small-molecule inhibitors and their clinical development. Curr Opin Pharmacol.

[18] Lundgren K, Zhang H, Brekken J (2009). BIIB021, an orally available, fully synthetic small-molecule inhibitor of the heat shock protein Hsp90. Mol Cancer Ther.

[19] Dickson MA, Okuno SH, Keohan ML (2013). Phase II study of the HSP90-inhibitor BIIB021 in gastrointestinal stromal tumors. Ann Oncol.

[20] Huang M, Zhang H, Liu T, Tian D, Gu L, Zhou M (2013). Triptolide inhibits MDM2 and induces apoptosis in acute lymphoblastic leukemia cells through a P53-independent pathway. Mol Cancer Ther.

[21] Nyåkern M, Tazzari PL, Finelli C (2006). Frequent elevation of Akt kinase phosphorylation in blood marrow and peripheral blood mononuclear cells from high-risk myelodysplastic syndrome patients. Leukemia.

[22] Yilmaz OH, Valdez R, Theisen BK, Guo W, Ferquson DO, Wu H, Morrison SJ (2006). Pten dependence distinguishes haematopoietic stem cells from leukaemia-initiating cells. Nature.

[23] Guo W, Lasky JL, Chang CJ (2008). Multi-genetic events collaboratively contribute to Pten-null leukaemia stem-cell formation. Nature.

[24] Breccia M, Alimena G (2010). NF-κB as a potential therapeutic target in myelodysplastic syndromes and acute myeloid leukemia. Expert Opin Ther Targets.

[25] Cilloni D, Martinelli G, Messa F, Baccarani M, Saglio G (2007). Nuclear factor κB as a target for new drug development in myeloid malignancies. Haematologica.

[26] Liu KS, Zhang Y, Ding WC (2012). The selective Hsp90 inhibitor BJ-B11 exhibits potent antitumor activity via induction of cell cycle arrest, apoptosis and autophagy in Eca-109 human esophageal squamous carcinoma cells. Int J Oncol.

[27] Calderwood SK, Khaleque MA, Sawyer DB, Ciocca DR (2006). Heat shock proteins in cancer: chaperones of tumorigenesis. Trends Biochem Sci.

[28] Mjahed H, Girodon F, Fontenay M, Garrido C (2012). Heat shock proteins in hematopoietic malignancies. Exp Cell Res.

[29] Jego G, Hazoumé A, Seigneuric R, Garrido C (2013). Targeting heat shock proteins in cancer. Cancer Lett.

[30] Neckers L, Workman P (2012). Hsp90 molecular chaperone inhibitors: are we there yet?. Clin Cancer Res.

[31] Chiosis G, Lucas B, Huezo H, Solit D, Basso A, Rosen N (2003). Development of purine-scaffold small molecule inhibitors of Hsp90. Curr Cancer Drug Targets.

[32] Braun T, Carvalho G, Fabre C, Grosjean J, Fenaux P, Kroemer G (2006). Targeting NF-kappaB in hematologic malignacies. Cell Death Differ.

[33] Kerbauy DM, Lesnikov V, Abbasi N, Seal S, Scott B, Deeq HJ (2005). NF-kappaB and FLIP in arsenic trioxide (ATO)-induced apoptosis in myelodysplastic syndromes (MDSs). Blood.

[34] Chapuis N, Tamburini J, Cornillet-Lefebvre P (2010). Autocrine IGF-1/IGF-1R signaling is responsible for constitutive PI3K/Akt activation in acute myeloid leukemia: therapeutic value of neutralizing anti-IGF-1R antibody. Haematologica.

[35] He Q, Li X, Zhang Z (2010). Overexpression of IGF-IR in malignant clonal cells in bone marrow of myelodysplastic syndromes. Cancer Invest.

[36] Mendoza MC, Er EE, Blenis J (2011). The Ras-ERK and PI3K-mTOR pathways: cross-talk and compensation. Trends Biochem Sci.

[37] Economopoulou C, Pappa V, Papageorgiou S (2010). Cell cycle and apoptosis regulatory gene expression in the bone marrow of patients with de novo myelodysplastic syndromes (MDS). Ann Hematol.

[38] Quesnel B, Guillerm G, Vereecque R (1998). Methylation of the p15(INK4b) gene in myelodysplastic syndromes is frequent and acquired during disease progression. Blood.

[39] Chen G, Zeng W, Miyazato A (2004). Distinctive gene expression profiles of CD34 cells from patients with myelodysplastic syndrome characterized by specific chromosomal abnormalities. Blood.

[40] Olnes MJ, Shenoy A, Weinstein B (2012). Directed therapy for patients with myelodysplastic syndromes (MDS) by suppression of cyclin D1 with ON 01910. Na Leuk Res.

[41] Böll B, Eltaib F, Reiners KS (2009). Heat shock protein 90 inhibitor BIIB021 (CNF2024) depletes NF-kappaB and sensitizes Hodgkin’s lymphoma cells for natural killer cell-mediated cytotoxicity. Clin Cancer Res.

